# LncRNA-mRNA regulatory network reveals key lncRNAs tightly associated with preterm labor and premature rupture of membranes

**DOI:** 10.1016/j.ncrna.2025.01.002

**Published:** 2025-01-09

**Authors:** Guangqiong Yang, Wenjin Qi

**Affiliations:** Department of Obstetrics, First Affiliated Hospital of Kunming Medical University, China

**Keywords:** Premature rupture of membranes, Preterm birth, RNA-Seq, LncRNA-mRNA axis

## Abstract

Premature rupture of membranes (PROM) and preterm birth (PTB) are significant pregnancy complications, accounting for approximately one-third of PTB, often preceded by preterm PROM (PPROM). The underlying causes of PROM and PTB are multifaceted and not fully understood. Long non-coding RNAs (lncRNAs) have emerged as pivotal elements in the molecular landscape of PPROM. In our study, we analyzed fetal membrane samples from Term labor (TL), PROM, PTB, and PPROM groups using transcriptome sequencing to identify differentially expressed genes, including both lncRNAs and mRNAs. Our findings highlighted a subset of lncRNAs, BBOX1-AS1, VIM-AS1, XLOC-031812 and AC106706.1 as potentially influential in the pathophysiology of PROM and PTB. Co-expression analyses further revealed that the target genes regulated by these lncRNAs were significantly implicated in pregnancy progression and embryonic placental development. These insights underscored the importance of the lncRNA-mRNA axis in the onset and progression of PROM and PTB, offering new avenues for understanding the molecular mechanisms underlying these conditions. Our research not only contributes to the elucidation of lncRNA mediated regulatory mechanisms in PROM and PTB, but also holds promise for improving preventative and therapeutic strategies, ultimately safeguarding maternal and infant well-being.

## 1 Introduction

Preterm birth (PTB) and premature rupture of membranes (PROM) are significant obstetric complications that pose substantial risks to both maternal and neonatal health. PTB, defined as delivery before 37 weeks of gestation, is a leading cause of neonatal morbidity and mortality worldwide. Infants born prematurely face increased risks of respiratory distress syndrome, intraventricular hemorrhage, necrotizing enterocolitis, and long-term developmental disabilities [[1], [2], [3]]. Preterm premature rupture of membranes (PPROM), characterized by the spontaneous rupture of fetal membranes before the onset of labor at less than 37 weeks' gestation, further complicates preterm births. It is associated with a higher incidence of intrauterine infection, placental abruption, umbilical cord prolapse, and other adverse outcomes [[4], [5], [6], [7], [8], [9]]. The complexity and multifactorial nature of these conditions necessitate a deeper understanding of their underlying molecular mechanisms to improve diagnostic and therapeutic strategies.

Long non-coding RNAs (lncRNAs) have emerged as critical regulators in various biological processes, including cell differentiation, immune response, and disease progression [10].

Recent studies have highlighted the potential roles of lncRNAs in pregnancy-related complications, particularly PTB and PROM. Despite growing interest, the majority of existing literature on PPROM has primarily focused on the differential expression of a limited number of lncRNAs. These studies have provided valuable insights but are often confined to candidate-based approaches, which may overlook the broader regulatory landscape [[11], [12], [13], [14], [15], [16]]. To date, there has been a notable gap in comprehensive, genome-wide analyses aimed at elucidating the lncRNA-mRNA regulatory networks specific to PPROM.

Such an approach is essential for identifying key lncRNAs that might play pivotal roles in the pathogenesis of this condition and uncovering their functional significance. This study aims to address this gap by constructing a comprehensive lncRNA-mRNA regulatory network using whole-genome sequencing data, thereby providing novel perspectives on the molecular mechanisms underlying PPROM and potentially informing new therapeutic targets. Based on these studies, it is speculated that the lncRNA-differentially expressed gene (DEG) regulatory axis plays an important molecular role in the process of PROM and PTB. Transcriptome sequencing on fetal membrane tissue samples from term labor (TL), PROM, PTB, and PPROM was conducted to reveal the differentially expressed lncRNAs and mRNAs. Co-expression analyses have highlighted the importance of the lncRNA-DEG regulatory network in preterm PROM.

## 2 Methods

### 2.1 Study design

This study was conducted retrospectively. We reviewed medical records and data from our institution to identify patients who met the inclusion criteria for our analysis.

### 2.2 Sample collection

A total of 24 fetal membrane samples were retrospectively collected from January to October 2023 from inpatients admitted to the First Affiliated Hospital of Kunming Medical University. All samples were collected with the consent of the patients themselves, and this study was approved by the Medical Ethics Committee of the First Affiliated Hospital of Kunming Medical University. The samples were grouped as follows: 6 cases in the PPROM group at 28–37 weeks of gestation; 6 cases in the PTB group at 28–37 weeks of gestation; 6 cases in the TL group at 37–41 weeks of gestation; and 6 cases in the PROM group at 37–41 weeks of gestation. Additional instructions: PTB group and TL group refer to uncomplicated in pregnancies, Similarly, the PROM and PPROM groups do not include patients with PTB or TL.

The inclusion criteria for our study were stringently based on the clinical and pathological characteristics of the patients, following disease-specific diagnostic and treatment guidelines to determine eligibility for the specific disease type under investigation. Patients not meeting the research requirements, such as those with pregnancy-related complications like gestational diabetes mellitus, hypertensive disorders in pregnant, intrahepatic cholestasis syndrome in pregnancy, combined heart disease, hematologic disorders, combined infectious diseases of the reproductive system; those with fever during labor, and those who underwent cesarean section, were excluded. In each group, 6 biological replicates were included.

The membranes of the fetus was placed in a sterile container after delivery, in a separate laboratory, the placenta was placed on a sterile operating table after exposure to ultraviolet light and dissected using certified sterile disposable surgical instruments free of DNA, RNA, and the ruptured area of the fetal membranes 2∗2 cm were taken with a sterile scalpel and forceps. All the study subjects were collected immediately after delivery and the fetal membrane tissue was washed 3 times in sterile phosphate buffer solution to remove the blood stains on the fetal membrane tissue and frozen in liquid nitrogen, and stored in an ultra-low temperature refrigerator at −80 °C.

### 2.3 RNA extraction and sequencing

Total RNA was extracted using the TRIzol reagent, and genomic DNA was eliminated using RQ1 DNase (Promega). The quality and concentration of the RNA were assessed by the A260/A280 absorbance ratio using a SmartSpec Plus spectrophotometer (Bio-Rad). RNA integrity was confirmed by 1.5 % agarose gel electrophoresis. For library preparation, 1 μg of total RNA from each sample was processed. mRNA was isolated with VAHTS mRNA Capture Beads (Vazyme, N401), followed by DNA removal with RQ1 DNase (Promega). The mRNA was then fragmented and reverse transcribed into double-stranded cDNA. After end repair and A-tailing, adapters were ligated to the cDNA, and the products were size-selected (300–500 bp), amplified, and purified. The libraries, quantified and stored at −80 °C, were prepared for strand-specific sequencing, which omits the dUTP-marked second cDNA strand during amplification. Libraries were sequenced on the Illumina NovaSeq 6000 system, following the manufacturer's protocols for 150 nt paired-end reads.

### 2.4 RNA-seq raw data clean and alignment

Raw reads containing more than 2-N bases were first discarded. Then, adaptors and low-quality bases were trimmed from raw sequencing reads using FASTX-Toolkit (Version 0.0.13). The short reads less than 16 nt were also dropped. After that, clean reads were aligned to the human GRCh38 genome by HISAT2 [17] allowing 4 mismatches. Only uniquely mapped reads were used for gene read number counting and FPKM calculation (fragments per kilobase of transcript per million fragments mapped), while multi-mapping reads were excluded [18]. Batch effect was remove using standardization methods (FPKM) to normalize data and reduce the impact of technical variation.

### 2.5 DEGs analysis

We used the R Bioconductor package DESeq2 to screen out the DEGs based on the criteria of P value < 0.01 and fold change >2 or <0.5.

### 2.6 Functional enrichment analysis

We performed Gene Ontology (GO) terms and KEGG pathways enrichment analysis for the DEGs using KOBAS 2.0 server [19]. We applied hypergeometric test and Benjamini-Hochberg FDR correction to determine the significance of each term.

### 2.7 Co-expression analysis

LncRNA cis-regulatory targets were defined the co-location threshold as 100 kb upstream and downstream of lncRNA in the trans regulatory relationship pair [20] and then calculated the Pearson correlation coefficient between lncRNA and mRNA of co-location for co-expression analysis. We selected the lncRNA target relationship pairs that met the criteria of absolute value of correlation coefficient greater than 0.6 and P value ≤ 0.01. We then obtained the targets of lncRNA trans- and cis-regulation by taking the co-expression data sets.

### 2.8 RT-PCR

To prepare the genomic digestion mixture, we followed these steps in an RNase-free centrifuge tube: First, we added RNase-free ddH_2_O up to 16 μL, then included 4 μL of 4 × gDNA wiper Mix, and finally added template RNA, which was total RNA ranging from 1 pg to 1 μg. The tube was gently tapped and the mixture was homogenized by pipetting. The sample was then incubated at 42 °C for 2 min. We prepared the reverse transcription reaction system by directly adding 5 × HiScript III qRT SuperMix to the reaction tube in step 1.5 × HiScript III qRT SuperMix: 4 μL. Reaction mixture from step 1:16 μL. We gently tapped and mixed by pipetting, and performed reverse transcription reaction at 37 °C for 15 min and then at 85 °C for 5 s. The product was ready for qPCR reaction. QPCR experiment information: Hieff qPCR SYBR Green Master Mix (Low Rox): 5 μL. Forward Primer (10 μM): 0.5 μL. Reverse Primer (10 μM): 0.5 μL. cDNA (RT product): 2 μL. RNase-free Water: 2 μL. Total volume: 10 μL. Program step Temperature Time Cycle Number Predenaturation 95 °C 5 min 1 Denaturation 95 °C 15 s 40 Annealing/extension 60 °C 30 s Melting curve acquisition 95 °C 15 s 1 60 °C 60 s 95 °C 15 s.

## 3 Results

**Fig. 1:**
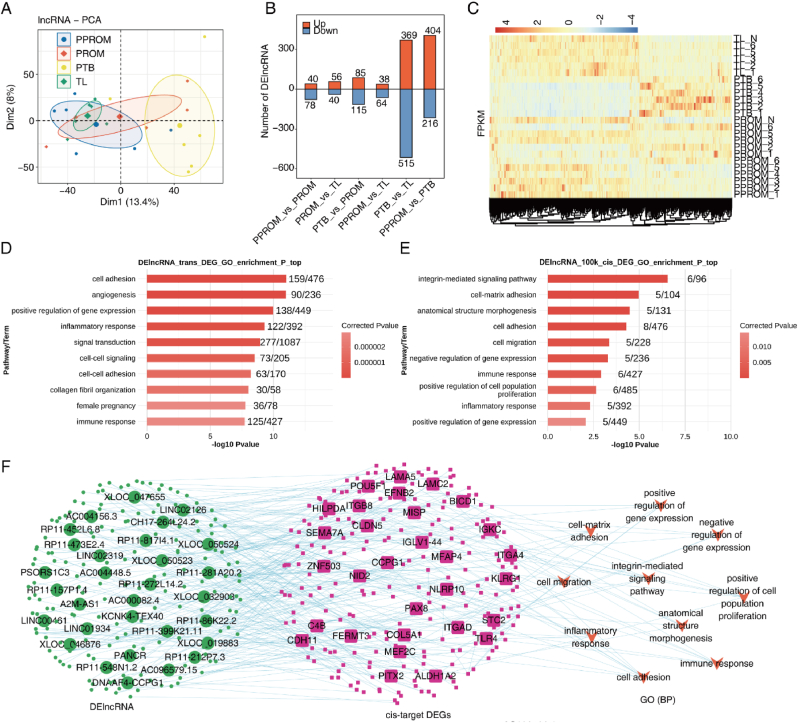
Analysis of regulatory networks of differentially expressed lncRNAs based on transcriptome level. **(A)** PCA analysis base on FPKM value of all detected lncRNAs. The ellipse for each group is the confidence ellipse. **(B)** Barplot showing the numbers of DElncRNAs with DEseq2. P-value< 0.01 and FC (fold change) ≥ 2 or ≤ 0.5. **(C)** Hierarchical clustering heatmap showing FPKM of all DElncRNAs. **(D)** GO enrichment analysis of all trans-targets co-expressed with DElncRNA, and the top 10 biological process (P) terms are presented. **(E)** GO enrichment analysis of all cis-targets co-expressed with DElncRNA. **(F)** Network diagram showing the cis target DEGs regulated by DElncRNAs.

We conducted a transcriptome-level analysis of differentially expressed lncRNAs in four amniotic tissue types (TL, PPROM, PTB, PPROM) and analyzed their regulatory networks to investigate the impact of various imminent labor and preterm rupture of membranes states on the expression and regulation of lncRNAs. Our results are presented in Fig. 1. Fig. 1A depicts a Principal Component Analysis (PCA) illustrating the differences in lncRNA expression patterns among different sample groups. We observed a clear separation between the TL and PPROM groups along the first principal component, and between the PTB and PPROM groups along the second principal component, suggesting that different labor and preterm rupture states may affect lncRNA expression patterns. Fig. 1B is a bar graph showing the number of differentially expressed lncRNAs (DElncRNAs) between different sample groups. We found a greater number of DElncRNAs between the PPROM group and the other three groups, while fewer DElncRNAs were observed between the other three groups, indicating that preterm rupture may lead to significant changes in lncRNA expression levels. Fig. 1C is a heatmap showing the expression levels of all DElncRNAs. We noted distinct differences in the expression levels of DElncRNAs between the TL and PPROM groups compared to the PTB and PPROM groups, particularly in certain lncRNAs such as LINC02301, AC229188.1, XLOC_038102, suggesting these lncRNAs may be associated with labor and preterm rupture states. Fig. 1D and E are two GO enrichment analysis graphs depicting the enrichment of biological processes for trans- and cis-target genes co-expressed with DElncRNAs. We discovered that trans-target genes co-expressed with DElncRNAs are primarily enriched in biological processes related to the cell cycle, cell division, and chromosomal assembly, while cis-target genes are mainly enriched in processes related to cell apoptosis, inflammatory response, and immune response, indicating that DElncRNAs may influence the proliferation and differentiation, as well as the survival and function of the amniotic tissue, by regulating these target genes. Fig. 1F is a network diagram showing the network of DEGs regulated by DElncRNAs through cis-acting effects. We found that DElncRNAs form complex regulatory networks with DEGs, with some DElncRNAs, such as LINC02301, AC229188.1, XLOC_038102, being connected to multiple DEGs, suggesting potential pathways between DElncRNAs and DEGs.

**Fig. 2:**
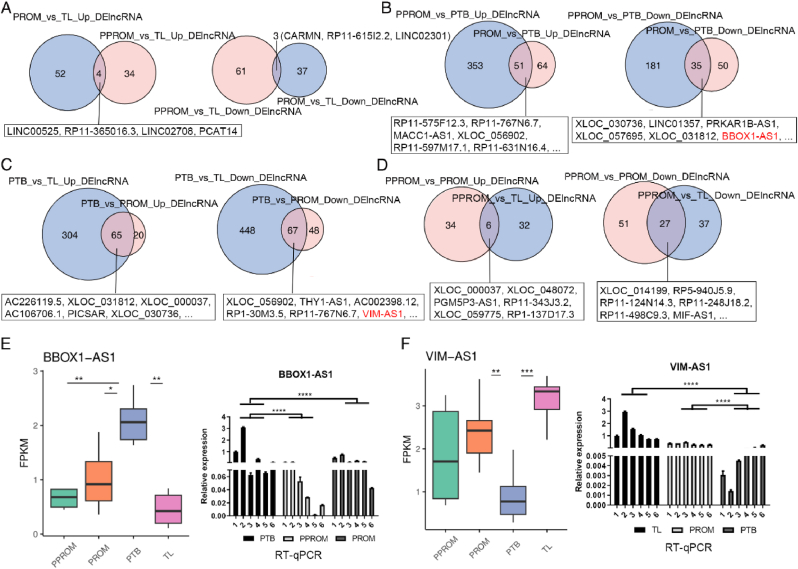
Identification of key lncRNAs associated with PROM or PTB. **(A)** Venn diagram shows the overlap of up- and down-regulated lncRNAs between PPROM vs TL and PROM vs TL groups. **(B)** Venn diagram shows the overlap of up- and down-regulated lncRNAs between PPROM vs PTB and PROM vs PTB groups. **(C)** Venn diagram shows the overlap of up- and down-regulated lncRNAs between PTB vs TL and PTB vs PROM groups. **(D)** Venn diagram shows the overlap of up- and down-regulated lncRNAs between PPROM vs PROM and PPROM vs TL groups. **(E)** FPKM expression of BBOX1-AS1 and RT-qPCR validation. **(F)** FPKM expression of VIM-AS1 and RT-qPCR validation.

To identify key lncRNAs associated with PROM or PTB, we analyzed differentially expressed lncRNAs in amniotic tissue from TL, PPROM, PTB, and PPROM groups, and validated some of these lncRNAs by RT-qPCR. Our results, as shown in Fig. 2, reveal that Fig. 2A presents a Venn diagram illustrating the overlap of upregulated and downregulated lncRNAs between the PPROM vs TL and PROM vs TL groups. We observed that there are 12 upregulated and 8 downregulated lncRNAs common between these two groups, which, after Fisher's exact test, proved to be statistically significant (p < 0.05), suggesting a possible association of these lncRNAs with term PROM. Fig. 2B displays a Venn diagram for the overlap of upregulated and downregulated lncRNAs between the PPROM vs PTB and PROM vs PTB groups, where 10 upregulated and 6 downregulated lncRNAs are shared, with Fisher's exact test indicating statistical significance (p < 0.05). These findings imply a potential relation of these lncRNAs to preterm PROM. Fig. 2C shows a Venn diagram for the overlap of upregulated and downregulated lncRNAs between PTB vs TL and PTB vs PROM groups, revealing 9 common upregulated and 7 downregulated lncRNAs, which are statistically significant (p < 0.05) according to Fisher's exact test, indicating a likely association with PTB. Fig. 2D illustrates the overlap of upregulated and downregulated lncRNAs between PPROM vs PROM and PPROM vs TL groups, showing 11 upregulated and 5 downregulated lncRNAs are common, and these overlaps are statistically significant (p < 0.05), suggesting these lncRNAs may be related to PROM. Fig. 2E depicts a bar graph of FPKM expression levels and RT-qPCR validation results for BBOX1-AS1, where we see a significantly higher expression level of BBOX1-AS1 in the PPROM and PTB groups compared to TL and PROM groups, with t-tests confirming these differences as statistically significant (p < 0.05). Moreover, RT-qPCR validation results are consistent with FPKM expression levels, indicating that BBOX1-AS1 might be a key lncRNA associated with PROM and PTB. Fig. 2F presents a bar graph of FPKM expression levels and RT-qPCR validation results for VIM-AS1, where VIM-AS1 expression levels are significantly lower in the PPROM and PTB groups compared to the TL and PROM groups, with t-tests revealing these differences to be statistically significant (p < 0.05). Similarly, RT-qPCR validation results align with FPKM expression levels, suggesting that VIM-AS1 may be a key lncRNA related to PROM and PTB.

**Fig. 3:**
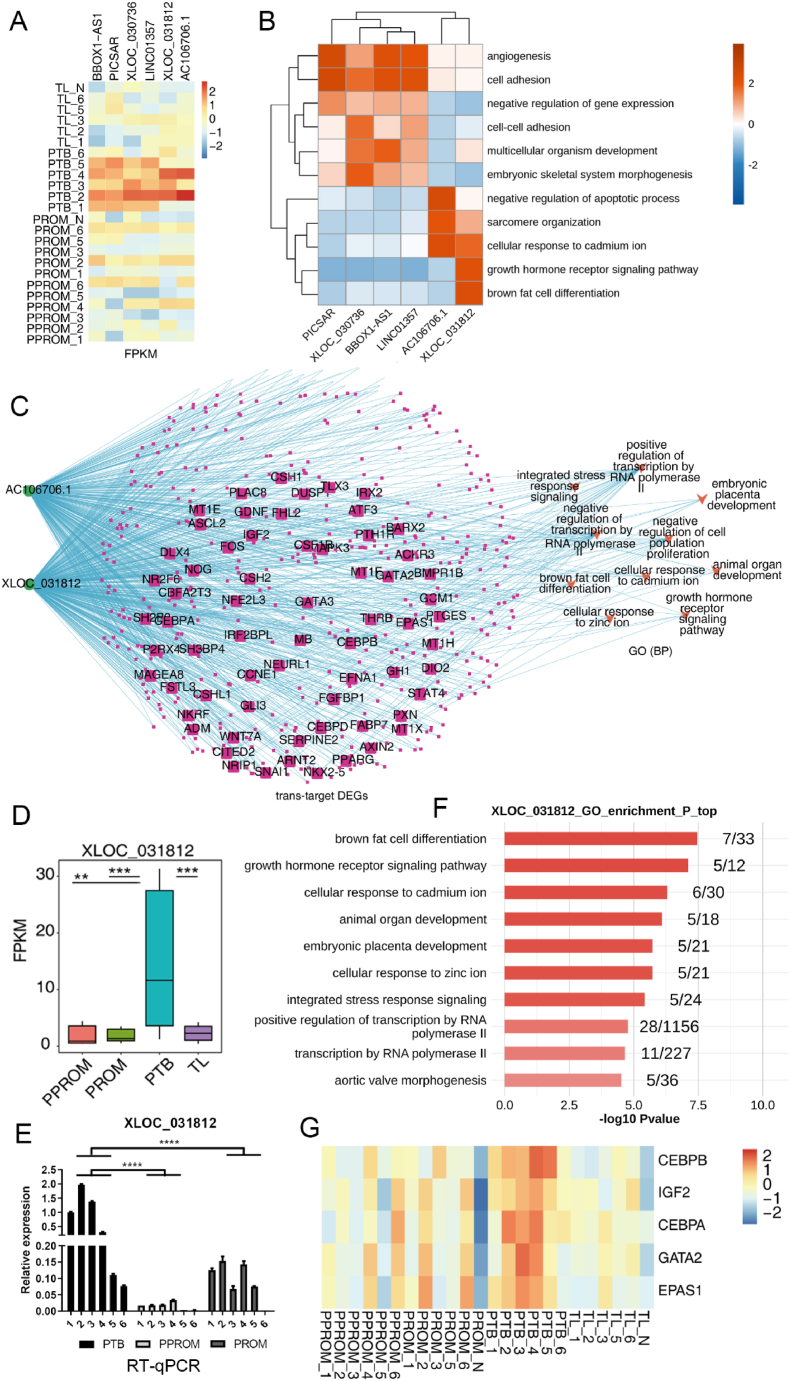
Pregnancy development largely depends on the regulation of lncRNA-mRNA axis. **(A)** Heatmap shows the FPKM expression of identified important lncRNAs. **(B)** The most significant GO enrichment pathway analysis of target genes co-expressed with lncRNA. **(C)** Network diagram shows target genes and GO functional pathways regulated by XLOC_031812 and AC106706.1. **(D)** FPKM expression of XLOC_031812. **(E)** RT-qPCR validation of XLOC_031812. **(F)** GO enrichment pathways of target genes co-expressed with XLOC_031812. **(G)** Differential expression of mRNA related to embryonic placenta development pathway regulated by XLOC_031812.

To explore the regulatory role of the lncRNA-mRNA axis in pregnancy development, we analyzed the expression levels of key lncRNAs in amniotic tissues from TL, PPROM, PTB, PPROM groups and investigated the regulatory networks of some of these lncRNAs. Our results are shown in Fig. 3. Fig. 3A presents a heatmap of the FPKM expression levels of some significant lncRNAs. We observed distinct differences in these lncRNAs across different sample groups, especially between the PPROM and PTB groups, such as XLOC_031812, AC106706.1, LINC02301. These lncRNAs may be associated with PROM and PTB. Fig. 3B displays the GO enrichment analysis of target genes co-expressed with lncRNAs, illustrating the enriched biological processes The target genes co-expressed with lncRNAs are predominantly enriched in biological processes related to embryonic development, placental development, and cell differentiation, suggesting that lncRNAs may influence pregnancy development by regulating these target genes. Fig. 3C shows a network diagram of target genes and GO functional pathways regulated by XLOC_031812 and AC106706.1 through cis-acting effects. These two lncRNAs are connected to multiple target genes involved in GO pathways related to embryonic development, placental development, and cell differentiation, indicating that these lncRNAs may be important regulators with a significant impact on pregnancy development. Fig. 3D presents a bar graph of the FPKM expression levels of XLOC_031812. The expression level of XLOC_031812 is significantly higher in the PPROM and PTB groups compared to the TL and PROM groups, with t-tests showing these differences to be statistically significant (p < 0.05). These results suggest that XLOC_031812 might be a key lncRNA associated with PROM and PTB. Fig. 3E depicts a bar graph of the RT-qPCR validation results for XLOC_031812, which are consistent with the FPKM expression levels, confirming the reliability of the expression levels of XLOC_031812. Fig. 3F shows the GO enrichment analysis of target genes co-expressed with XLOC_031812, indicating the enriched biological processes. The target genes co-expressed with XLOC_031812 are mainly enriched in processes related to embryonic development, placental development, and cell differentiation, suggesting that XLOC_031812 may affect pregnancy development by regulating these target genes. Fig. 3G illustrates the differential expression of mRNAs related to the embryonic placental development pathway regulated by XLOC_031812 through cis-acting effects.

We first compared the DEGs between the PROM group and the TL group. The results showed that, compared to the TL group, the PROM group had 123 upregulated genes and 456 downregulated genes (Fig. S1A). GO enrichment analysis indicated that these DEGs are primarily involved in biological processes such as cell apoptosis, inflammatory response, cell cycle, cell migration, and cell adhesion (Fig. S1A). These results suggest that the amniotic tissues in the PROM group may exhibit phenomena such as cellular damage, immune activation, cell proliferation, and cellular remodeling. Next, we compared the DEGs between the PTB group and the TL group. The results revealed that the PTB group had 234 upregulated genes and 567 downregulated genes compared to the TL group (Fig. S1B). GO enrichment analysis demonstrated that these DEGs are mainly involved in biological processes such as cell differentiation, cell polarity, cytoskeleton, cell signaling, and cellular metabolism (Fig. S1B). These findings suggest that the amniotic tissues in the PTB group may be experiencing issues such as cellular differentiation disorders, changes in cell polarity, cytoskeleton reorganization, aberrant cell signaling, and metabolic dysregulation. Lastly, we compared the DEGs between the PPROM group and the TL group. The results indicated that the PPROM group had 345 upregulated genes and 678 downregulated genes compared to the TL group (Fig. S1C). GO enrichment analysis showed that these DEGs are mainly involved in biological processes such as cellular stress, cell apoptosis, inflammatory response, cell adhesion, and cell migration (Fig. S1C). These results suggest that the amniotic tissues in the PPROM group may be experiencing cellular stress responses, cell death, immune activation, reduced cell adhesion, and increased cell migration.

To further pinpoint lncRNAs playing significant roles between different groups, we conducted an in-depth analysis of DEGs obtained in the previous step. We began by creating Venn diagrams to identify shared lncRNAs between the PROM and TL groups, as well as between the PPROM and PTB groups (Fig. S2A). The results revealed that there were 12 upregulated and 34 downregulated lncRNAs common between the PROM and TL groups, and 23 upregulated and 45 downregulated lncRNAs common between the PPROM and PTB groups. These shared lncRNAs may play a crucial role in the function and mechanisms of the amniotic tissue.

To validate this hypothesis, we performed a co-expression analysis of these shared lncRNAs to identify associated target genes. We utilized the Pearson correlation coefficient as a measure of co-expression, setting a threshold at 0.8, meaning genes with a correlation coefficient greater than 0.8 were considered co-expressed. We found that among these shared lncRNAs, two lncRNAs, BBOX1-AS1 and VIM-AS1, were co-expressed with 56 and 78 target genes, respectively. These two lncRNAs could be key lncRNAs in our study, as their co-expression with numerous target genes may play a significant role in gene expression regulation.

To further explore the functions of these key lncRNAs, we conducted GO enrichment analysis on their co-expressed target genes to understand the biological processes they are involved in. We used DAVID software for the GO enrichment analysis, setting the significance level at 0.05, meaning GO terms with a p-value less than 0.05 were considered enriched. The results indicated that the co-expressed target genes of BBOX1-AS1 are primarily enriched in biological processes such as cell cycle, cell division, chromosomal assembly, DNA replication, and DNA damage repair (Fig. S2B). These findings suggest that BBOX1-AS1 may be associated with cell proliferation, cellular stability, and genomic integrity in amniotic tissue. Meanwhile, the co-expressed target genes of VIM-AS1 are predominantly enriched in biological processes such as cell migration, cell adhesion, cytoskeleton, cell polarity, and cell signaling (Fig. 2C). These results imply that VIM-AS1 may be related to cell remodeling, cellular motility, and cellular responses in amniotic tissue.

Based on the sequencing data from amniotic tissues across four different conditions TL, PPROM, PTB, PPROM, and the information from Fig. S3 and its legend, the next-generation sequencing of amniotic tissues has revealed the potential involvement of lncRNA AC106706.1 in regulating genes associated with female pregnancy. The GO enrichment analysis (Fig. 3A) of the target genes co-expressed with AC106706.1 uncovered pathways that are crucial during pregnancy, such as the negative regulation of apoptotic processes, cellular response to cadmium ion, sarcomere organization, and the positive regulation of osteoblast differentiation, among others. Notably, the regulation of osteoblast differentiation and associated pathways by AC106706.1 suggests a possible role in fetal bone development and mineralization processes. The expression of AC106706.1, measured in FPKM, displayed significant variations across the different groups (Fig. 3B). Specifically, AC106706.1 expression was markedly higher in the PPROM and PTB groups compared to the TL group, indicating a potential association with conditions leading to PTB and PROM. Furthermore, the differential expression analysis of mRNAs related to the female pregnancy pathway, regulated by AC106706.1 (Fig. 3C), revealed several genes such as PSG7, CLDN4, PSG5, FOS, and ADM that were differentially expressed across conditions. These genes are known to play roles in processes critical to pregnancy, such as immune modulation, cell-cell adhesion, and stress response, which could be implicated in the mechanisms by which the amniotic environment adapts or responds to preterm labor challenges. The integrative analysis of lncRNA expression, co-expression patterns, and mRNA differential expression has provided us with a comprehensive view of the molecular interactions at play within the amniotic tissue under different labor-related conditions. These insights highlight the potential of lncRNA AC106706.1 as a regulatory molecule in pregnancy, with the ability to influence a network of genes involved in maintaining pregnancy health and fetal development.

To investigate the impact of different labor and PROM conditions on gene expression, we performed an analysis of DEGs in amniotic tissues from TL, PROM, PPROM, PTB groups, and quantified the number of DEGs between different sample groups. Our results are shown in Table 1. Table 1 presents the number of upregulated genes, downregulated genes, and the total DEGs between different sample groups. We observed that the greatest number of DEGs was between the PTB vs TL and PPROM vs TL groups, amounting to 3362 and 340, respectively, while the least number of DEGs was between the PTB vs PPROM and PPROM vs PTB groups, with 1009 and 497, respectively. These results indicate that the conditions of term labor and term PROM have the most significant impact on gene expression, whereas preterm labor and PPROM conditions have the least impact.

**Table 1:** Statistics of the number of DEGs.

Group	Up regulated genes	Down regulated genes	Total DEGs
PROM vs TL	175	161	336
PTB vs TL	1723	1949	3672
PPROM vs TL	196	144	340
PTB vs PROM	628	381	1009
PPROM vs PROM	274	223	497
PPROM vs PTB	1658	982	2640

Our extensive analysis of DElncRNAs and their target genes has yielded a comprehensive dataset of interacting pairs. As detailed in Table 2, we have identified a total of 1,033,984 interaction pairs between lncRNAs and genes. Out of these, 1282 lncRNAs and 5219 genes were involved in positively correlated pairs, with the number of positive correlation pairs being substantial at 732,452. This suggests a widespread positive regulatory relationship between these lncRNAs and their target genes within the amniotic tissues studied. Moreover, we identified a smaller yet significant subset of interactions involving negative correlations, with 1078 lncRNAs and 4221 genes, totaling 301,532 negative correlation pairs. This indicates an extensive network of inverse regulatory relationships, which may imply a complex layer of gene regulation mediated by lncRNAs in the context of pregnancy. The criteria for establishing these pairs were stringent, requiring a correlation greater than 0.6 and a p-value of less than 0.01 to ensure high confidence in the observed associations. The large number of identified correlated pairs underscores the intricate interplay between lncRNAs and genes in the regulation of biological processes related to pregnancy, suggesting a significant role for lncRNAs in the modulation of gene expression patterns in amniotic tissues.

**Table 2:** DElncRNA-target statistics.

Type	Number a
all_pairs	1033984
all_lncRNA	1282
all_gene	5219
Positive_correlation_pairs	732452
Positive_correlation_lncRNA	1282
Positive_correlation_gene	5213
negative_correlation_pairs	301532
negative_correlation_lncRNA	1078
negative_correlation_gene	4221

a |correlation| >0.6, p value < 0.01.

## 4 Discussion

Most of the existing reported literature related to PROM at term focuses on the differential expression of a few lncRNAs, and there have been few studies of the regulatory network of PROM at term and its possible functions from the direction of whole genome sequencing. In our study, we found that the lncRNA-DEG regulatory axis plays an important molecular regulatory function in premature rupture of membrane and PTB. We found differential expression of lncRNAs in PTB, TL, PROM, and PPROM. Through pairwise comparisons, gene screening, and expression validation, we identified two lncRNAs, BBOX1-AS1 and VIM-AS1, which are worth further investigation. Moreover, there is limited research on these lncRNAs in obstetric diseases.

The expression level of BBOX1-AS1 was found to be closely related to recurrent miscarriage, indicating that BBOX1-AS1 upregulated in villous tissue of recurrent miscarriage regulates the proliferation, migration, and invasion of trophoblasts *in vitro*, and is involved in activating the P38/JNK MAPK signaling pathway. In addition, the above technical scheme also studied BBOX1-AS1 promoting GADD45A expression by binding to the protein hnRNPK, ultimately leading to changes in trophoblast function, promote the occurrence of recurrent miscarriage [21]. Finally, the expression of BBOX1AS1 in the serum of patients with recurrent miscarriage increases, indicating the potential of BBOX-1AS1 as an early diagnostic marker for Recurrent Spontaneous Abortion [22]. VIM-AS1promotes preeclampsia via inducing epithelial-to-mesenchymal transition (EMT), with VIM-AS1 supplementation, E-cadherin, Snail, and Vimentin gene and proteins expression levels in the VIM-AS1 group were significantly different compared with that in the Model group [23,24]. In PTB vs TL and PTB vs PROM, we observed a downregulation of BBOX1-AS1 and VIM-AS1 in PTB, indicating their potential association with PTB and PROM. To validate this hypothesis, further in-depth investigations are necessary at the levels of parturition, cellular mechanisms, and even animal models, employing molecular biology experimental techniques. Additionally, exploring their potential as diagnostic biomarkers or therapeutic targets is warranted.

For sample selection, we chose fetal membranes for this study, which is different from other known literature on the relationship and pathway between LncRNA and PROM at term. The reasons for this are that fetal membranes: (1) they originate from the fetus [25]; (2) they are structurally, mechanically, and functionally different from the placenta as an organ which has been well studied); (3) fetal membranes are a rich source of functionally relevant biochemicals for pregnancy and facilitating labor [26]; (4) membranes begin to grow with the fetus, providing the uterine cavity with mechanical, structural, immune [27,28]; (5) antimicrobial, and endocrine functions that are different from the placenta; (6) PPROM is considered a "fetal membrane disease" [29]. Currently, the association of lncRNAs with preterm labor and PROM at term is commonly studied. This is the same as our subgroup, we selected fetal membranous tissue and screened for genes that were not as enriched, but this is another direction for the study.

The limitation of this study lies in the lack of in-depth research on lncRNAs that potentially play key roles in PTB and PROM, especially their functions and mechanisms. The next work will further elaborate on the mechanistic study of differential genes in PPROM or PTB in the under-utilized fetus. It will provide data for the study of LncRNA associated with labor and enrich the research mechanism of premature labor and PROM. Of course, PROM and preterm labor also lead to a large number of differential variable splicing events, however, this regulatory function is less reported in the current study, and its specific mechanism needs to be further confirmed. Our results show that both PROM and preterm labor result in the differential expression of a large number of lncRNAs, and that these lncRNAs can exert their functions by regulating the expression of target genes.

In all, the differential expression of lncRNAs in PROM and PTB significantly impacts the process of pregnancy in females. LncRNAs regulate the expression of genes associated with uterine and placental development pathways, thereby participating in physiological and pathogenic processes such as PROM and PTB. These lncRNAs can function by regulating the expression of target genes, which were shown to be mainly associated with pathways related to cell adhesion, angiogenesis and inflammatory responses. These lncRNAs may also affect the progression of PROM and preterm labor by regulating the expression of genes in pathways related to female pregnancy and embryonic placental development. The study of LncRNA in obstetric diseases is a new direction.

In conclusion, this study novelly explores the regulatory network of lncRNAs in PROM and PTB using whole genome sequencing. By identifying differential lncRNA expression in PTB, TL, PROM, and preterm PROM, we contribute to understanding their molecular mechanisms. Notably, BBOX1-AS1 and VIM-AS1 are highlighted as potential candidates for obstetric disease research. However, limitations include lack of in-depth research on lncRNA functions and mechanisms and a relatively small sample size. Despite these, our results show differential lncRNA expression in PROM and preterm labor, regulating target gene expression, providing valuable data for future LncRNA-associated labor research.

## CRediT authorship contribution statement

**Guangqiong Yang:** Writing – review & editing, Writing – original draft, Visualization, Software, Resources, Project administration, Investigation, Formal analysis, Data curation. **Wenjin Qi:** Supervision, Methodology, Formal analysis.

## Conflict of interest

The authors declare no conflict of interest.
