# Supplementary material for: LncRNA-mRNA regulatory network reveals key lncRNAs tightly associated with preterm labor and premature rupture of membranes
Source: Noncoding RNA Res. 2025 Jan 9;11:273–80. doi: 10.1016/j.ncrna.2025.01.002 (PMC12015680; doi:10.1016/j.ncrna.2025.01.002)
Supplement: Multimedia component 1 [file mmc1.doc]

(1):ii

**
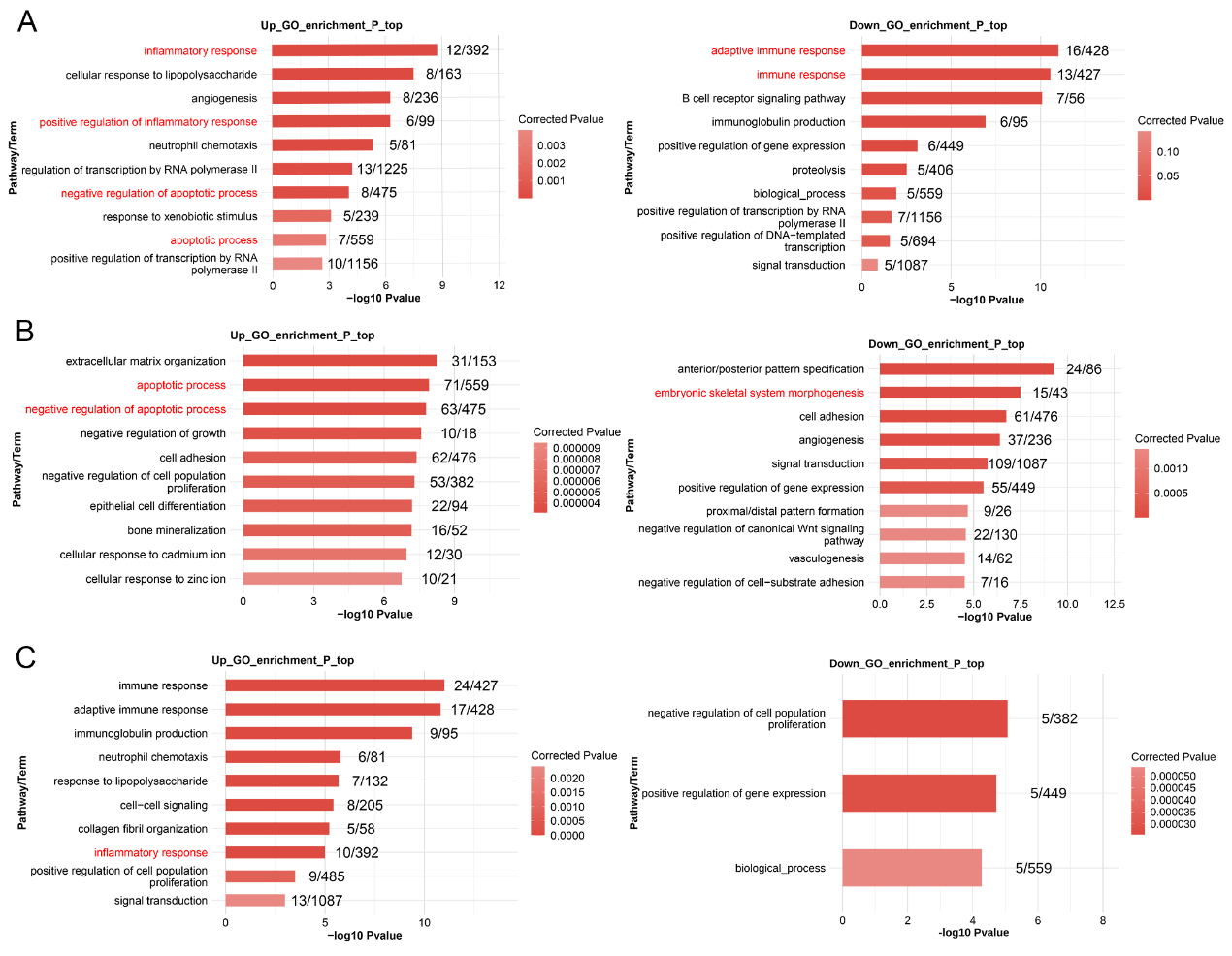
**

**Fig.S1 GO enrichment analyses of DEG between groups. (A)** shows the GO-P results of up- and down-regulated DEGs from PROM vs TL. **(B)** shows the GO-P results of up- and down-regulated DEGs from PTB vs TL. **(C)** shows the GO-P results of up- and down-regulated DEGs from PPROM vs TL.


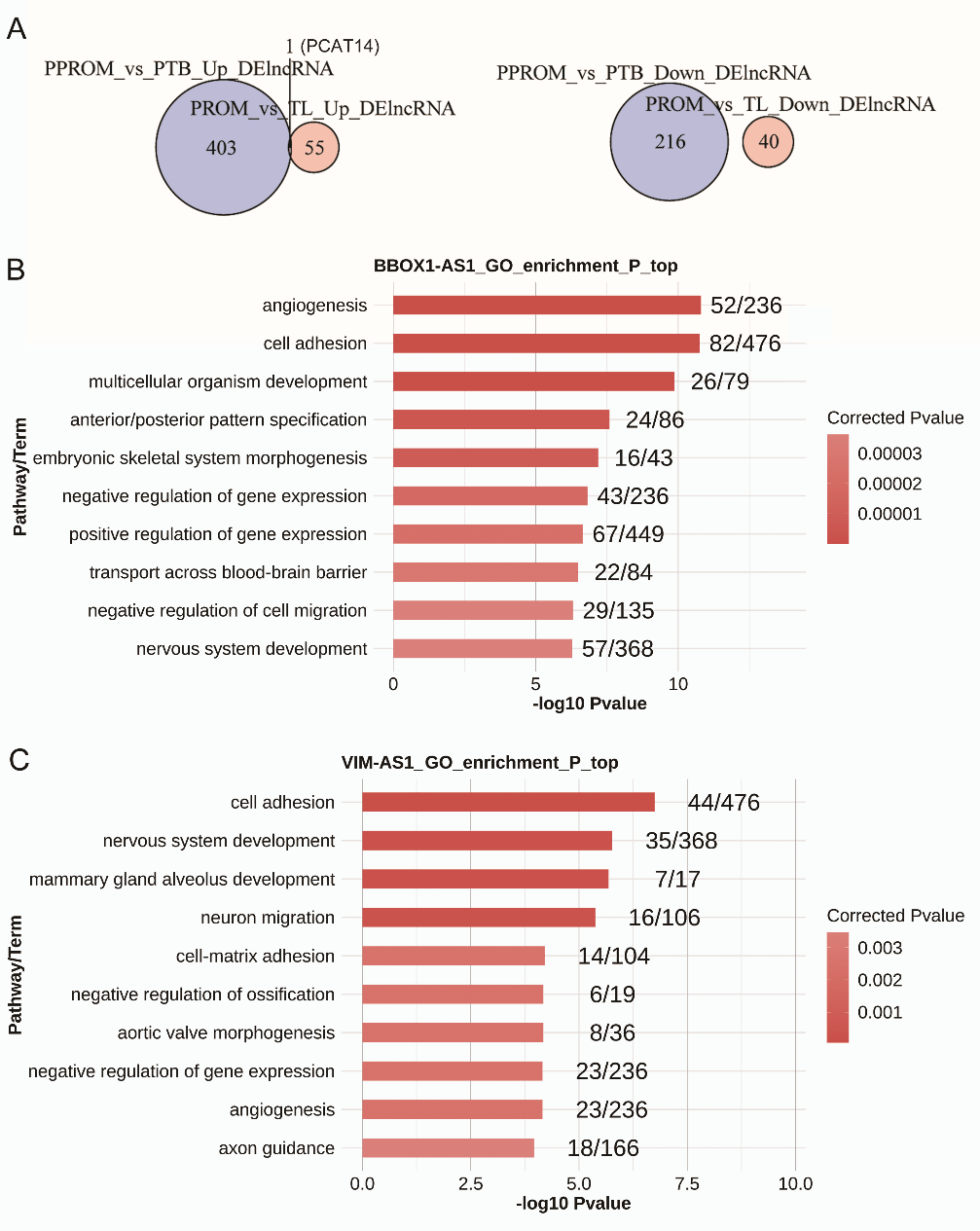


**Fig.S2 Identification of key lncRNAs.** **(A)** Venn diagram shows the overlap of up- and down-regulated lncRNAs between PPROM vs PTB and PROM vs TL groups. **(B)** GO enrichment pathways of target genes co-expressed with BBOX1-AS1. **(C)** GO enrichment pathways of target genes co-expressed with VIM-AS1.


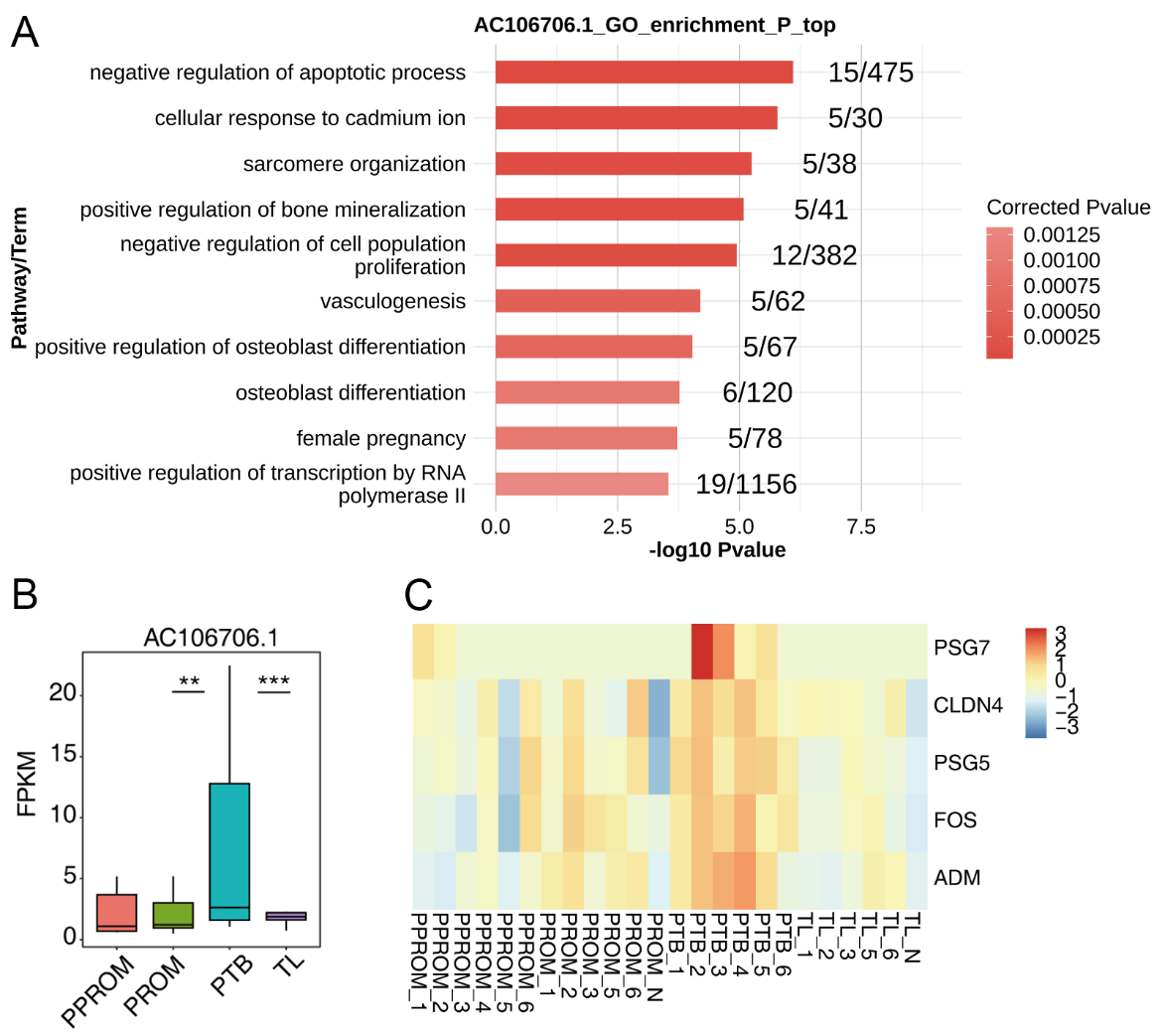


**Fig.S3 LncRNA may be involved in female pregnancy by regulating mRNA expression. (A)** GO enrichment pathways of target genes co-expressed with AC106706.1. **(B)** FPKM expression of AC106706.1. **(C)** Differential expression of mRNA related to female pregnancy pathway regulated by AC106706.1.

|  |  |  |  |
| --- | --- | --- | --- |
|  |  |  |  |
|  |  |  |  |
|  |  |  |  |
|  |  |  |  |
|  |  |  |  |
|  |  |  |  |

able 2. DElncRNA-target statistics

|  |  |
| --- | --- |
|  |  |
|  |  |
|  |  |
|  |  |
|  |  |
|  |  |
|  |  |
|  |  |
|  |  |
